# *Pseudomonas aeruginosa*: An Uncommon Cause of Antibiotic-Associated Diarrhea in an Immunocompetent Ambulatory Adult

**DOI:** 10.1155/2020/6261748

**Published:** 2020-09-02

**Authors:** Ryan T. Hoff, Ami Patel, Alan Shapiro

**Affiliations:** Department of Medicine, Division of Gastroenterology, Advocate Lutheran General Hospital, Park Ridge, USA

## Abstract

*Pseudomonas aeruginosa* is an opportunistic Gram-negative pathogen known to cause enterocolitis in children, amongst other types of infections. *Pseudomonas aeruginosa* has been widely reported as a cause of antibiotic-associated diarrhea in adult immunocompromised hosts. We present an 81-year-old previously healthy female as the first reported case of *Pseudomonas aeruginosa* antibiotic-associated diarrhea in an immunocompetent host in the United States.

## 1. Introduction

*Pseudomonas aeruginosa* is an opportunistic Gram-negative pathogen known to cause enterocolitis in children and nosocomial infections, including hospital-acquired pneumonia and catheter-associated urinary tract infections (UTIs). Rarely, *Pseudomonas aeruginosa* causes antibiotic-associated diarrhea in immunocompromised hosts, but this has not been reported in immunocompetent ambulatory adults.

## 2. Case Presentation

**Figure 1:**
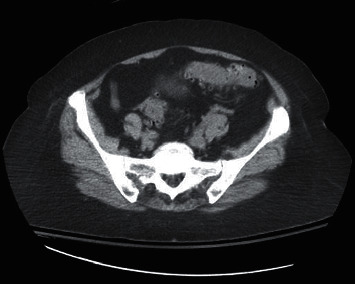
CT abdomen and pelvis without contrast, showing mild inflammation surrounding the sigmoid colon. Diverticulosis is present.

**Figure 2:**
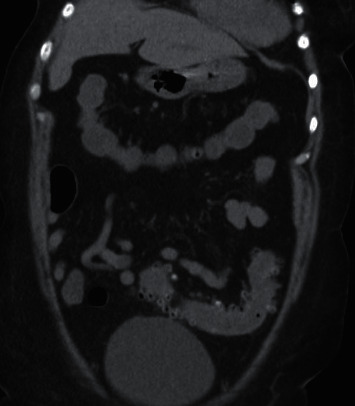
CT abdomen and pelvis without contrast, showing mild inflammation surrounding the sigmoid colon. Diverticulosis is present.

An 81-year-old previously healthy woman presented to the emergency department (ED) with diarrhea for 2 days. A week prior to symptom onset, she completed a 17-day course of ciprofloxacin for a UTI, with resolution of dysuria. She described frequent, watery, nonbloody bowel movements with nausea and emesis. Her last colonoscopy 5 years prior showed only diverticulosis. She was treated with intravenous saline and ondansetron and discharged. The next day, she developed bloody bowel movements associated with weakness and mild lower abdominal pain. With ongoing diarrhea, she returned to the ED. Physical exam showed very mild left lower quadrant abdominal tenderness, without rebound or guarding. Laboratory studies showed white blood cell count 11,800 cells/mcL, potassium 3.3 meq/dL, lactate 1.0 mmol/L, and lipase 67 units/L. Electrolytes and liver enzymes were otherwise normal. *Clostridioides difficile* (*C. difficile*) stool toxin PCR was negative. CT abdomen and pelvis without contrast showed diverticulosis and mild inflammation surrounding the sigmoid colon (Figures 1 and 2). Empiric ciprofloxacin and metronidazole were started, and the patient was admitted to the medical floor. Over the next 3 days, she gradually improved. Stool culture grew *Pseudomonas aeruginosa*, with absence of normal fecal flora. Sensitivities showed resistance to ciprofloxacin and levofloxacin and sensitivity to all other antibiotics tested. Given her clinical improvement, antibiotics were discontinued after 7 days.

## 3. Discussion

We report a case of *Pseudomonas aeruginosa* as a cause of bacterial colitis. Our patient's recent long course of antibiotic therapy likely increased her susceptibility to this infection via loss of normal fecal microbiota, leading to a loss of competitive exclusion and diminished colonic barrier protection. While the episode of bleeding and abdominal discomfort could be manifestations of ischemic colitis, this would not explain the diarrhea or stool culture results.

*Pseudomonas aeruginosa* has been reported as a cause of enterocolitis in children and may affect healthy children [1]. Outbreaks in nurseries and infant wards have been reported [2, 3]. The most common risk factor is antibiotic use [1], though it also occurs in association with conditions that cause immunosuppression, including acute leukemia [4]. *Pseudomonas* enterocolitis in children may be severe, with bloody diarrhea, prolonged fever, markedly elevated inflammatory markers, and necrotizing enterocolitis, or mild with watery diarrhea in the absence of prolonged fever [1]. *Pseudomonas aeruginosa* has also been reported to colonize the gastrointestinal tract in patients with cystic fibrosis [5]. A combination of both host and pathogen factors likely contribute to this organisms' ability to cause overt infection [6].

*Pseudomonas aeruginosa* has been reported to cause rare cases of hospital-associated diarrhea in adults. A Korean study by Kim et al. described seven cases of nosocomial antibiotic-associated diarrhea due to *Pseudomonas aeruginosa*, as diagnosed by stool culture [7]. One patient was also diagnosed with *C. difficile* infection. All probable offending antibiotics were either carbapenems or cephalosporins, with an average duration of therapy of 13 days prior to onset of diarrhea and median onset at day 19 of hospitalization. Unlike our patient, fluoroquinolones were not implicated in this series. Sensitivity testing demonstrated that all strains of *Pseudomonas* were resistant to the previously administered antibiotics, suggesting a loss of competitive inhibition may have occurred. In one case series from a public hospital in Brazil, 14 patients admitted to the intensive care unit had a stool culture positive for *Pseudomonas aeruginosa*, although six of those patients also tested positive for *C. difficile* [8]. Alternative causes are important to consider, and patients with suspected *Pseudomonas* colitis should undergo testing for *C. difficile*.

An Australian study by Adlard et al. of 23 patients with diarrhea for more than 1 week found *Pseudomonas aeruginosa* in 20 cases without any other associated cause for diarrhea [9]. Of these patients, 19 were immunosuppressed due to age, comorbid illness, or medication therapy [3]. After pretreating rats with clindamycin, inoculation with *Pseudomonas aeruginosa* led to prolonged fecal shedding of the bacterium as well as ulceration and necrosis of the terminal ileum in 75 percent of the animals [9].

In cases of antibiotic-associated *Pseudomonas aeruginosa* enterocolitis, discontinuation of the antibiotic may be sufficient for clinical improvement. However, patients with ongoing clinically significant diarrhea would likely benefit from antimicrobial therapy, which may aid in recovery and facilitate restoration of normal bowel flora. Improvement has been reported with ciprofloxacin therapy [10]. However, the role for antimicrobial therapy in *Pseudomonas aeruginosa*-associated diarrhea has not been studied; further investigation is warranted.

To our knowledge, this is the first reported case of acute postantibiotic diarrhea caused by *Pseudomonas aeruginosa* in an immunocompetent ambulatory host in the United States. Previous reports of *Pseudomonas* colitis have primarily involved immunosuppressed or hospitalized patients and children. This pathogen may represent an important diagnostic consideration in patients presenting with diarrhea following antibiotics, with negative *C. difficile* stool testing. The role for antibiotic therapy in *Pseudomonas*-associated diarrhea warrants further research.
